# How Technologies Can Support Self-Injury Self-Management: Perspectives of Young Adults With Lived Experience of Nonsuicidal Self-Injury

**DOI:** 10.3389/fdgth.2022.913599

**Published:** 2022-06-29

**Authors:** Kaylee Payne Kruzan, David C. Mohr, Madhu Reddy

**Affiliations:** ^1^Department of Preventive Medicine, Center for Behavioral Intervention Technologies, Feinberg School of Medicine, Northwestern University, Chicago, IL, United States; ^2^Department of Informatics, University of California, Irvine, Irvine, CA, United States

**Keywords:** digital mental health, digital intervention, mHealth, self-injury, self-harm, mobile app, self-management

## Abstract

**Background:**

There is growing interest in the design of digital interventions to improve conditions for young people who engage in high-risk behaviors, like nonsuicidal self-injury (NSSI). However, few studies have focused on how young people self-manage NSSI, or their existing, and historic, use of technologies to support their goals related to NSSI behavior change. Such an understanding has the potential to inform the design of digital interventions that meet this population's unique needs.

**Objectives:**

This study aims to (a) understand the self-management practices of young adults who engage in NSSI, (b) explore how they currently use technologies for self-injury self-management, and (c) identify the ways they can envision an app-based technology supporting their self-management.

**Methods and Materials:**

Twenty young adults (aged 18–24) with lived experience of NSSI, and who were not currently enrolled in therapy, were recruited from online venues. Participants completed baseline measures to assess mental health and NSSI characteristics, followed by a virtual 1-h semi-structured interview where they were invited to share their experience of self-management, their goals, and their thoughts on supportive technology. Interview scripts were transcribed and analyzed *via* thematic analysis.

**Results:**

Themes and sub-themes are organized under two broad domain areas: (1) How young adults self-manage NSSI thoughts and behaviors and (2) Opportunities and challenges for digital interventions to assist young adults in their recovery process. We found that young adults had varied experiences with, and goals related to, NSSI. Participants reported a lack of effective strategies to reduce NSSI urges and a desire for an app-based technology to track patterns and deliver personalized suggestions for self-management. Participants reported existing use of technologies as part of self-management, as well as early information and support seeking for NSSI online.

**Conclusions:**

This study contributes a greater understanding of young people's experiences with self-injury, their self-management practices, and their desire to engage with technology. Our findings highlight the need for design flexibility in developing digital interventions that support individual goals, unique presentations of NSSI, and needs at different phases of recovery. Implications for the design of highly personalized and relevant digital interventions to address NSSI are discussed.

## Introduction

Nonsuicidal self-injury (NSSI) is a growing public health concern, affecting 13% of young adults and 17% of adolescents worldwide (1, 2). For many NSSI can be a way to manage distress or attain other functional outcomes focused on establishing intra- or inter-personal homeostasis. Though NSSI occurs without suicidal intent, it commonly co-occurs with suicidal ideation and is a leading risk factor for future suicidal behaviors and attempts, marking a need for early and effective intervention (3–6).

Many adolescents and young adults who engage in NSSI do not access professional treatment (7–10). Research has shown barriers to treatment, including cost, accessibility, and attitudinal barriers such as stigma, fears around disclosure, or not recognizing the need for treatment, are common (8, 11, 12). Digital interventions offer a promising alternative to reach young people not engaged in treatment since they may circumvent some barriers experienced, and are aligned with young adults' preference for autonomy (13–15). Indeed, many young people report receptivity to digital mental health interventions, such as mobile apps (16–19).

Most research at the intersection of technology and NSSI has focused on how young people exchange information and support around mental health and NSSI online, through forums and social media (20–23). Collectively, this work has contributed to an understanding of NSSI recovery including young people's needs, concerns, goals, and motivations for NSSI and NSSI-related help seeking.

Research on digital interventions for NSSI is more limited but growing. Several apps and web-based treatments have been developed and undergone preliminary tests of their efficacy. For example, two apps designed as adjuncts to face-to-face treatment produced promising reductions in NSSI. DBT Coach, an app for adults with borderline personality disorder (BPD), promotes dialectical behavior therapy (DBT) skills generalization and practice in daily life. In a small pilot trial that introduced the app to 16 adults with BPD and self-harm after 6 months of standard DBT treatment, app use was associated with reductions in urges to self-harm, NSSI frequency, and subjective distress after 3 months use (24). Another app, BlueIce, focused on skills from cognitive behavioral therapy and DBT, was designed as an adjunct to face-to-face therapy for young people who self-harm (25). In a 12-week open trial with 44 adolescents, 73% of users reported having stopped or decreased self-injury. Qualitative feedback from participants following the trial suggest that among perceived benefits of the app were facilitating mood tracking, trigger identification, and distraction (17). Other web-based programs mirroring the structure of manualized treatments have incorporated apps as a component of the intervention and shown similarly promising results, including reductions in NSSI (26). Digital interventions based on more experimental models, like conditioning and expressive writing, have produced mixed evidence on efficacy for NSSI reductions (27, 28).

In addition to efficacy trials of digital interventions, several studies have focused on the types of digital tools young people would find helpful in their NSSI recovery (29–31). In an interview study with 15 adolescent girls who met criteria for NSSI disorder (32), participants reported receptivity to smartphone interventions for NSSI and described the need for easy-to-implement emotion regulation strategies and coping strategies for managing urges. Participants also reported difficulties reaching out for support, and interest in customization, and psychoeducation around alternatives to NSSI.

Another study involved 6 young adults with lifetime suicidal ideation or self-harm in interviews to understand their experiences of NSSI urges and how a technology may be useful (33). Findings suggested that an app-based intervention would be most useful if it allowed users to store things that would make them feel good, and improve mood in moments when they were experiencing an NSSI urge. Participants also wanted to be able to share or exchange helpful strategies with others who have similar experiences. Subsequent co-design workshops by this same research group were held with adolescents and clinicians to ideate on features of a future app. The workshops resulted in a low-fidelity prototype called the “well-being tracker,” which allowed for mood tracking, customization, and brief interventions or suggestions to be used between face-to-face appointments (31).

In general, these studies point to several desired features for future digital interventions, including brief on demand coping skills, psychoeducation, social connection, and opportunities to gain self-knowledge about patterns and address common comorbidities or other life challenges. However, less is known about the specific ways these features should be instantiated within an app to best support young people's natural use of technology and their self-management patterns. Additionally, the generalizability of these findings to populations who are not currently treatment-engaged is unknown as both studies focused on young people who are actively in contact with mental health services. The current study was designed to address these gaps and detail the experiences and needs of young adults who are not currently engaged in treatment for NSSI, and the ways a digital tool may be able to better support them in their recovery.

### The Current Study

Designing technologies to fit within individuals' daily lives and habitual patterns requires a nuanced understanding of their current self-management practices, challenges, and technology-use. The present study aims to provide a nuanced examination of young adults' lived experience of NSSI and directly solicit feedback on their imagined use of technologies to support their goals. The broad aims are: (1) to understand the self-management practices of young adults who engage in NSSI, (2) explore their use of technologies to support their efforts toward NSSI and mental health management and (3) identify ways young adults could (or could not) envision using technology (e.g., apps, etc.) to support them in managing their NSSI.

*Note:* We use the term self-management deliberately as we did not want to convey any expectation or make assumptions about participants' desire to *change* their NSSI behavior. Given that many participants endorsed coping strategies as part of self-management practices, we use the two terms interchangeably to refer to shorter- and longer-term efforts to improve or maintain well-being. This includes but is not limited to management of NSSI. Similarly, when we discuss participant goals in the findings section this term was used to describe anything participants would like to work toward regarding their NSSI or mental health. In the interviews, participants were provided with this broad description and were able to offer alternatives if the term “goal” did not resonate with them.

## Method

### Recruitment

Participants were recruited from online venues between May and October of 2021. This included emailing individuals who were part of several research registries (e.g., Research Match, Center for Behavioral Intervention Technologies registry, and Cornell Research Program on Self-Injury and Recovery) and posting online study advertisements through social media platforms (e.g., Facebook and Reddit). All recruitment materials included a clickable link to a description of the study for interested individuals which included access to the online eligibility screener. For this study, inclusion criteria included: (1) engaging in NSSI on 2 or more days in past month, (2) between ages of 18–24 years old, (3) own a smartphone, and 4) US Citizen. Exclusion criteria were guided by our aim to design a digital self-management intervention and safety considerations. These included (1) severe mental health diagnoses (e.g., psychotic or bipolar disorders), for which a standalone digital intervention would not likely be appropriate; (2) severe suicide risk, including suicidal ideation with a plan and intent to act; and (3) current engagement in psychotherapy, since we were interested in meeting the needs of a population currently uninterested or underserved in existing services. Eligible participants received an email to set up a remote interview time, which also contained a link to the online consent and baseline survey with additional items on mental health and NSSI history. Interviews were conducted by the first author over zoom, were under 1 h, and were audio recorded and transcribed prior to analysis. All recruitment and study procedures were approved by the university's Institutional Review Board.

### Measures

The interview script was co-developed by the authors and contained questions related to (1) managing mental health and self-injury, (2) technology use in mental health self-management, and (3) imaged use of an app. For a full list of questions please see the Supplemental Materials. Prior to the interview, participants completed a brief survey to assess their NSSI history and mental health comorbidities. NSSI history was assessed *via* the Alexian Brothers Assessment of Self-Injury – Short Form (ABASI-SF), a self-report measure that assesses the specific methods and frequency of NSSI, as well as medical severity, impulsivity, and experience of their NSSI behaviors (34). NSSI disclosures was assessed *via* the Nonsuicidal Self-injury Assessment Tool (NSSI-AT), a self-report questionnaire focused on NSSI characteristics. Only the disclosure subscale was administered which contained items related to perceptions of others' awareness of their NSSI, as well as disclosure experiences (e.g., Someone knows that I intentionally hurt myself and has had a conversation with me about it) (35). All participants additionally completed the PHQ-9 (36), a nine item self-report assessment of depressive symptoms, as part of our risk management procedure.

Mental health diagnoses were assessed at eligibility *via* self-report measure containing nine common and/or exclusionary diagnoses (e.g., “Has a mental health provider or physician ever diagnosed you with…”) To assess experience with mental health treatment, participants additionally responded to two questions “Have you ever seen [Are you currently seeing] a therapist, counselor, psychologist for mental health treatment?”

### Analysis

Thematic analysis, as described by Braun and Clarke (37), was used to code and analyze all interview data. This process included six steps including: data familiarization, systematic identification of individual codes, grouping codes into preliminary themes, reviewing and refining themes to reduce overlap, defining and naming the final themes common across whole dataset, and selecting examples from the data to accurately illustrate each theme. After several rounds of iterative open coding, the code structure was discussed among authors for face validity and conceptual clarity, and the remaining transcripts were coded. Codes were organized into a candidate set of themes which was refined to remove redundancy and retain only highly prevalent themes. All coding occurred in Dedoose software. Descriptive statistics including frequencies, means, and standard deviations were run in IBM Corps statistical analysis program SPSS version 27 (38).

### Participant Safety

Procedures were developed to ensure participant safety throughout the study. During the baseline assessment, suicidality was assessed by self-report using item #9 of the PHQ-9. If participants score ≥1 on the PHQ-9 item 9, they received a suicide item (item 9) from Beck Depression Inventory (2nd edition). If they scored above a 1 on both they were called within 24 h and issued the Columbia Suicide Severity Rating scale. There was no formal assessment of severity of mental health symptoms or suicide during the interview. However, all participants were informed that their participation was voluntary and that they could pause or discontinue the interview at any time without consequences. They were also informed that they could skip questions if they felt uncomfortable.

One participant scored at or above a “1” on the 9th item of the PHQ-9, was issued the BDI-II and scored above a 2 which indicates elevated risk. This participant was therefore administered the full Columbia Suicide Risk Assessment, followed by safety planning, prior to the interview. The participant was deemed to be at low risk based on clinical assessment, so we proceeded with the interview. Additionally, all participants were provided contact information, including phone numbers and links to crisis resources including the National Suicide Prevention Lifeline, the Crisis Text Line, and the Trevor Project.

### Participant Characteristics

Participants were 20 young adults with recent (past month) engagement in NSSI. All participants were between the ages of 18 and 25 (*M*_age_ = 20.7, SD = 2.2; Mode = 19). Most identified as female (65%; *n* = 13), 2 (10%) identified as male, 3 (15%) identified as non-binary or third gender, 1 (5%) identified as transgender, and 1 (5%) self-described as She/They. Most participants self-identified their race as White (80%; *n* = 16), 2 (10%) as Asian, 1 (5%) as more than one race, 1 (5%) as Black or African American.

Most participants reported their first NSSI behavior around the age of 13 (*M*_age_ = 13.45, SD = 2.98, Mode = 11) and having engaged in NSSI on around 5 days the past month (*M* = 5.21 days, SD = 3.46, Mode = 4). The most common forms of NSSI were cutting (*n* = 14, 70%), scratching to the point of bruising or bleeding (*n* = 17, 85%), hitting oneself (*n* = 15, 75%), banging head or limbs against something (*n* = 14, 70%), and preventing wounds from healing (*n* = 12, 60%). Nine participants had made a prior suicide attempt (45%). Eighteen participants reported that they wanted to stop or decrease self-injury, while two disagreed. One of the two agreed that NSSI was a problem, and the other did not. While all participants were not currently engaged in mental health treatment, most participants had at one time seen a therapist, counselor, or psychologist for mental health treatment (*n* = 16, 80%) and 60% indicated that someone knows about their self-injury. Self-reported comorbid conditions are reported in Table 1.

**Table 1:** Participant characteristics.

	***N* (20)**	**%**
**Mental health comorbidities**
Depression	15	75
Anxiety	14	70
Obsessive compulsive disorder	4	20
Posttraumatic stress disorder	5	25
Eating disorder	5	25
Borderline personality disorder	2	10

## Results

Below we present themes and subthemes organized under two broad section headings focused on: (1) How young adults self-manage NSSI thoughts and behaviors and (2) Opportunities and challenges for digital interventions to assist young adults in their recovery process.

### How Young Adults Self Manage NSSI

Participants' reflections on their self-management of NSSI thoughts and behaviors, and related mental health symptoms, resulted in three themes: (1) differing goals and motivations, (2) strategies for self-management, and (3) technology as part of the self-management process.

### Differing Goals and Motivations

Participant goals and motivations varied in ways that reflected how they understood and related to their NSSI thoughts and behaviors. Some participants identified specific goals related to their experience of NSSI ranging from complete cessation of the behavior to reductions in the frequency and severity of injuries. Others described broader goals related to improved mental health and well-being, such as the desire to acquire new strategies to reduce NSSI as well as symptoms of comorbid mental health conditions like depression, anxiety, and disordered eating. Commonly participants endorsed more than one goal, identifying both short-term and long-term goals, that would help them to feel better in the short term and more confident in their ability to navigate difficult emotions or situations overtime.

Stopping NSSI was the most common long-term goal reported by participants, with just two exceptions, while short-term goals were more personalized and varied across participants. Among those reporting intentions to work toward reductions in NSSI severity or frequency, harm-reduction techniques were often endorsed, including using different (less damaging) methods, replacement behaviors, and engaging in safer NSSI practices. Reflecting on both a long and short-term goal, one participant describes:

“*Well, this is a general long-term one, but I would like to stop. It*'*s not something I want to do my whole life. I view it as an addiction, and that*'*s not a good mindset to have about anything. More current or short-term, I guess less frequently or less severely. There*'*s some behaviors that are arguably worse than others. If I have to engage in some harmed behavior, I would rather it be something that doesn*'*t leave a permanent mark vs. something that does” (P5)*.

Many participants described using “*alternatives that aren*'*t as self-destructive”* (P19) to reduce the physical impact of NSSI, without stopping the behavior entirely. Another participant described wanting to minimize the amount of harm done (or minimize the risk of unintentional harm) saying: “*obviously [I] wanna just stop completely. But more specifically, I would say reduce the severity of the cuts… Maybe just put it less deep, maybe surface level cuts, if anything” (P12)*.

Though many participants described the use of less-severe methods or replacement behaviors as part of their NSSI self-management, they were not often seen as a viable solution long-term. On this, participant 16 commented “*The ice trick was not helpful. The rubber-band trick is tricky…you can leave marks and … welts if you try hard enough… the rubber band trick might be like a slippery slope kind of thing. I don*'*t know if that's helpful or not.”* Not all participants had positive experiences with harm reduction techniques. One participant described that she would tell herself that she “*would only do just a little bit”* but then found it “*really hard to stop”* once she started (P10). In sum, participants described varying degrees of success and comfort with cessation and harm-reduction as part of their personal histories.

*Importantly, e*ven among those that described strong intentions to reduce NSSI over time, there was a great deal of ambivalence expressed. Many participants felt the behavior would be too difficult to stop, often because they had been injuring for a long time. One participant described: “*I've been dealing with these thoughts since I was 13, so, it's about seven years now, you just kind of get tired… It doesn't get better, so why bother not doing it?”* (P20) Vague intentions to stop were also common such as one participant who described wanting to “*kick the habit”* but having “*no small specific goals at the moment… it's like biting your nails. It's like, ‘Well, I do want to stop. I just haven't put the stuff that tastes bad on my nails, or I haven't like worn the caps. I haven't done anything”* (P11).

Finally, NSSI was seen as a coping strategy for many participants reflecting the nuance of NSSI as part of a more complex mental health history. The idea that any change in NSSI was possible, or desired, did not resonate with these participants experiences. Rather, these participants felt they needed to resolve preexisting mental health symptoms, or to feel confident in using other effective strategies before they were willing to consider addressing NSSI. Reflecting this, one participant said: “*I'm sort of cool with it. I think there's a bigger problem, and until I deal with the bigger problem, I don't feel like dealing with the symptoms would be beneficial”* (P13). Embedded in many of these responses was the clear functionality of the behavior, and a fear, in some cases, of losing NSSI as a method to manage distress.

### Strategies for Self-Management

Most participants endorsed several strategies they had used, or tried, to achieve their mental health or NSSI goals. Outside of harm reduction techniques, participants described avoidance strategies reporting mixed success. Activities aimed at distracting participants from intense NSSI urges or thoughts, including reaching out to friends to talk about something unrelated, watching TV, or doing “mundane tasks” like organizing their room were common. Others qualified that they needed to engage in activities that fully occupied their mind or hands, often citing video games as an example of an activity that did both: “*keeping your hands occupied is important. That's why I like playing video games as opposed to watching TV because when you're playing video games you have to be holding a controller, or using a mouse, you don't have idle hands”* (P5). For others more vigorous physical activity like boxing or running were perceived to be a helpful way to discharge overwhelming emotions or energy and made them tired, and therefore, less likely to act on urges.

Though distraction was a go-to strategy for participants who characterized NSSI thoughts and urges as momentary, some participants described their thoughts and urges as too intense and persistent to manage with acute in-the-moment activities. For these participants staying “constantly busy” and actively avoiding down-time when they may be able to act on those thoughts, felt necessary.

While less common, several participants mentioned cognitive and behavioral approach strategies like talking back to, or challenging, negative thoughts, engaging in pro and con exercises (e.g., “*I start listing off reasons why I shouldn't do it” P20)* or the use of distress tolerance skills from DBT, like TIP (temperature, intense physical exertion, and paced breathing) or STOP (stop, take a step back, observe, proceed mindfully). Those who endorsed approach strategies often described being introduced to them through past therapy or through searching for skills online.

Though all participants described at least one way that they managed their NSSI urges and behavior, the most consistent sentiment participants expressed was their frustration by the lack of effective strategies they found to cope with NSSI. As a representative example, one participant described:

“*I've tried loads of different things. I don't really do therapy … but I have in the past. That didn't really help. You know, relaxation techniques and things like that, but that really didn't help either. So, breathing exercises and grounding just, neither of those really seem to work. I'd listen to music and things like that and sometimes I play video games and stuff and that kind of helps a little bit. But it's like, it's just a distraction really. It doesn't really solve the problem.” (P20)*.

This desire for more effective coping strategies that resolve both short-term distress and provide longer-term relief in the form of self-efficacy was pervasive.

### Technology as Part of the Self-Management Process

Technology was embedded within participants' self-management practices and a means to engage in coping strategies, to access information, and to connect with community. Participants reached for their phone, tablet, or computer to connect with others for support, distract in moments of distress, or seek content that could shift their mood. Activities like boxing or running were often done while listening to evocative music through headphones. In fact, several participants described curating playlists to match the intensity of their emotions, or to invoke safe mental states.

In addition to technology being part of specific self-management practices, internet-based technologies were also used by participants to seek information about self-injury. For example, one participant described: “*when I was a teenager, and I was first struggling with it…. I didn't understand why I was doing this and what I was going through”* so she went online to get “*more general knowledge about it”* (P15). Another participant described searching for NSSI alternatives on social media: “*I would go through the tag and read what people would do as an alternative. That used to be a big, big thing where these posts would go around that would be like, ‘Instead of doing this, put an ice cube on your arm, or drawn on yourself with marker”’* (P7). Such information-seeking often led participants to connect with peers with shared experiences through online communities.

While some participants described current use of online communities to connect, most participants reflected on the benefits of these spaces when they were younger, with their utility declining with time. For example, one participant described “*when I was younger, I'd use Tumblr a lot to post and reach out to people with similar situations and see – I think, maybe, they wouldn't give advice, but knowing I'm not alone was helpful, having a sense of community”* (P19). Rather than discontinuing use, some participants noted that their use patterns changed as their needs shifted over time: “*I do use them now but not really in the same way”* (P2).

Some participants identified that their needs shifted as they developed a greater capacity to manage their mental health. For example, one participant describes “*When I was younger. I know I did in middle school quite a bit. But the more that I was able to kind of work through … on my own, and make strong, deep bonds with people, I found that actually having the person-to-person connection has been more beneficial to me”* (P18). By contrast, for others, it was less about a shift in their needs and more an increased awareness of the harmful qualities of online communities that prompted them to disengage or use them less as they entered young adulthood. Indeed, many participants commented on the potential for negative experiences in these online spaces. For example, one participant notes:

“*I think eventually I realized that it was just very triggering. Reading about people's sort of own struggle with self-harm just backfired and made me think about it all the more frequently and also gave me new ideas of things to try and that kind of stuff. So, eventually, I was like, I can't. I can't do that anymore”* (P5).

In sum, while technology was part of participants' self-management process, our participants described use for information and support in the early phases of their NSSI, with a change in use as their needs evolved.

### Potential for Digital Intervention

Findings up to this point provide critical context for understanding how to support young adults in managing NSSI and mental health. In the remaining sections, we shift focus to participants' ideation on the use of technology to help them work toward their NSSI goals. Below we report on two major themes with multiple sub-themes: (1) What participants would like to receive through the technology, and (2) How they would like to engage with the technology, including perceived facilitators and barriers to engagement and usefulness.

#### What Young Adults Would Like to Receive

Here, we describe three desired components of a digital intervention for young adults who self-injure, including: (1) safe connection to social support, (2) tracking patterns for improved self-knowledge and personalized recommendations, and (3) varied support for mental health and NSSI-specific needs.

##### Safe Connection to Social Support

Participants expressed a desire to connect with others who have shared experiences with NSSI, but in a way that differed from prior experiences in online communities or support forums. While connecting with peers online helped address issues of loneliness and validated their struggles, they reported trouble leveraging this beneficial support to make concrete changes in their self-management in daily life.

The usefulness of hearing peer stories, particularly strategies or activities peers engaged in to meet their goals, was unanimously supported. For instance, a participant described that hearing peer stories “*help[s] a lot, especially when they struggle, they not only struggle with self-injury, but other things like, certain trauma or something like that. But it really feels like you can connect with the other person even though you're not talking to them”* (P20). For this participant part of the value of hearing from peers was in recognizing her story and struggles in theirs. Knowing that peers shared common demographics, or mental health histories (e.g., trauma) made their advice more credible. On this, one participant described his connection to peer stories like this:

“*if we have a lot of similar characteristics, I tend to listen to their stories more closely. And I tend to find a lot of their solutions are very personal to them. And I assume that if we share a lot of the same characteristics, maybe they could be personal for me, too. So, I try to see some very specific things that people have done… something that [is] smart and intuitive and knew what's gonna work on a 19-year-old is not gonna work on a 40-year-old and vice versa” (P12)*.

Participants imagined that social support could be delivered in an app format to capitalize on the benefits of peer support (e.g., validation, experiential knowledge), while also promoting a sense of autonomy in their efforts toward better health and protecting against the harms. One participant described a suggestion feature where they could share and learn about different strategies that have worked for peers with similar characteristics: “*If you had them, the people with similar experiences, input their suggestions into the app, and then have those suggestions be reviewed, and then go through a process where they can be spit back out at people who need suggestions. To better manage the stuff that they have going on”* (P15). Building on a similar idea described another participant that a network of users could generate a bank of content for on-demand suggestions or support.

In general, participants felt it was important for any future digital intervention to incorporate the benefits of peer support in a format that could reduce the harms associated with open online forums, and enable autonomous interaction with the app.

##### Understanding and Tracking Patterns for Improved Self-Knowledge and Personalized Recommendations

Participants hoped that the app could help them understand and track patterns, which could increase self-knowledge and be used for self-diagnostics. Participants who did not have a clear understanding of why they engaged in NSSI, thought it would be useful for the app to track (passively and actively) emotions, behaviors and contexts uniquely associated with their self-injury in order to receive personalized insights or recommendations. When comparing his experience receiving generic solutions offered *via* online sources, one participant described the promise of an app with tracking functionalities like this:

“*If there would be a complex step-by-step process on what you can do, I would actually appreciate it a lot. Because I find these solutions, like the ones I mentioned before, they don't really solve the root of the problem. They're temporary fixes. And if you actually wanna defeat something like this, you have to really get to the root of the problem and work on very specific solutions. I don't know what they are at the moment. I can't really help myself, of course. But if there was something that could help me do that, I would appreciate it.”* (P12)

As reflected in the above quote, many participants expressed a desire to respond proactively to urges and a need to identify “root problems,” which we understood to mean things that made individuals vulnerable to NSSI in the first place. There was an expectation that such tracking would lead to more specific and personalized strategies, that they could then use and gather additional data on. On this, one participant that the app could both teach, and log the use of, new strategies: “*Different coping skills where you'd have to do them over time to see if they would be effective for you. So, then, it would help a person keep track to see if it worked for them”* (P14). Participants were also willing to put in effort to get good insights if they could help them navigate distressing future situations:

“*If it had the option of putting more things in, you get to, obviously, learn more, like if it had a option of, briefly, what you did that day. If there was just certain things you could click like stayed home, went to work – things like that – I feel like it would more, too, to realize how your moods are affected by the activities you do throughout the day because then you'd realize like, “Oh, when I go to work, I tend to be more on the angry side.” (P19)*

Several participants mentioned using the “notes” feature on their phone to keep track of things that helped them when they experienced urges and saving ideas for future use. For example, participant 7 said: “*Sometimes I try and write down what I'm feeling. I guess it's in the place of just telling it all to somebody and really just getting all my feelings out somewhere.”* These participants imagined an augmented app feature that allowed them to record notes in the service of detecting patterns and improving insights. Importantly, though most participants recognized the value of being able to track patterns significant to their own goals and recovery, there is a need to be careful with this feature as some participants reported that self-competition, disappointment or shame could result from seeing declines in progress over time. This is discussed in more detail later.

Finally, participants wanted the app to have on-demand activities that could help them de-escalate the intensity of their thoughts, emotions, or urges. One participant described: “*You could have some sort of activity within the app that takes 10, 15, 20 minutes, however long, because I think for a lot of people… oftentimes the urge will pass within 20, 30 minutes, an hour, so if they get over that hump, then they're fine” (P5)*. She goes on to clarify “*that doesn't work for everyone, but for some people, I think it does. So, having some sort of engaging activity that you can do easily, just laying in bed or sitting at your desk, for however long you need, I guess, until you're done, would be helpful” (P5)*.

As the quote highlights, activities need to be varied in length and easily completed. For others it was more important that the app could connect them to activities that would generate or shift specific emotional states.

##### Varied Support for Mental Health and NSSI-Specific Needs

Nonsuicidal self-injury is often cyclical and long gaps can exist between episodes of NSSI behavior. This fact was reflected in participants' desire for the app to contain content and strategies for periods of more frequent and intense NSSI, as well as content and strategies that could help them with a broader range of mental health outcomes and goals. As one participant described that it should not be a “*sadness app where I go when I'm sad. It could also just be somewhere that I also associate when going when I'm in a good place”* (P13). The inclusion of resources related to other mental health symptoms, was particularly compelling for those participants who understood their NSSI behaviors to be a symptom of a broader problem. Participant 7 describes,

“*I would like to probably engage with it every day including days when I'm not feeling negative. I think in that case, that would be when it would be the most effective. Because sometimes, it feels really, really silly to be in such an awful, terrible mood, and downloading an app. A lot of times … they'll be like, “Hey, we haven't seen you for a week. How you doing?” And it's like, “I'm doing awful.” And then in that case, when I'm feeling very bad, I think I'm more resistant to help and reframing my thoughts and thinking positively. So, I think if it was something more like a routine, if I did it when I felt very good as well as when I felt very bad, then it would probably be more effective for me.”*

In addition to diversifying content based on mental health needs, participants desired for the app to support them in meeting shorter- and longer-term goals which meant it should have resources for imminent distress as well as maintenance. One participant notes the need for features aimed at maintenance like this*: “Usually, if I have the urge, then I will engage in self-injury. I try to avoid having the urge because once I do, it doesn't feel like there's much I can do to get rid of the urge without actually acting on it”* (P5). Similar reflections were noted by other participants who identified their goal as not having the urge to self-injure in the first place.

#### How Young Adults Would Like to Engage

Finally, in addition to describing what would be helpful in an app to support NSSI self-management, participants provided insight on specific features or interactions that would either encourage or dissuade them from app use. We report on two key sub-themes related to: (1) facilitators and (2) barriers to use of an app in the following sections.

##### Facilitators to Use of an App

Common facilitators to app use included interactions that respected and reinforced autonomy and features that provided incentives and positive reinforcement. Participants consistently described the need to feel autonomy when interacting with the app, and in their mental health management. While they appreciated the idea of the app making suggestions for how to manage NSSI, they needed to feel like the decision to engage in new strategies was ultimately theirs. Indeed, participants described the imagined app as assisting in self-regulation, and that a fully automated app would allow them to express themselves more honestly and freely: “*I think definitely talking to a robot or something along those lines would help me feel a lot more confident expressing myself because I don't have to worry about how it's gonna be received. I could just get the solutions, be very honest to myself and to the person behind it, and move forward from there”* (P12). Another participant similarly commented:

“*I feel on an app it's more personal. It's yourself making you do it kind of thing, but if it's not on the app and like someone else is telling you, ‘Hey, maybe you should…’ for me, it gives me more of like, why? Now I really don't want to, because someone's saying like, ‘Hey, go do this thing’ But like, in my own head, like, Hey, maybe you should, or being pushed by the app to make me think positively”* (P4).

The idea of an app utilizing data that participants input themselves (either actively through survey, or passively through sensors), made receiving suggestions more palatable than if they would have received that information from another person who did not have their exact set of experiences. Though many participants recognized the value in having peers or professionals involved, they overwhelmingly expressed a desire to use this app on their own and described concerns about privacy and safety that might make them disengage. One participant said “*I can imagine maybe it to be nice to have a mental health professional accessible. I can't really think of what I would use it for, but I can imagine it being probably helpful. But I don't think I'd like to have other people involved in my thing”* (P13).

Incentives were also mentioned as a way to motivate users and encourage app use. Participants described that the app's benefit needed to be apparent in the initial interactions to prevent early disengagement. As a solution, participants recommended an incentive structure where they would be provided with positive reinforcement when engaging in activities that were aligned with their goals (e.g., a number of days without NSSI). One participant described, “*I always like things that monitor your progress and sort of celebrate whenever like…you know, it'd be cool to have an app … that celebrated every day you were clean, and sort of kept track and that didn't make you feel guilty when you relapsed or whatever. Like, something to encourage you to feel proud of yourself for not self-harming” (P20)*. However, others were skeptical about the effects of positive reinforcement, saying that this type of feedback could come across as silly or patronizing if not designed with a clear attention to detail. Indeed, participant comments were highly varied on the types of messages they would like to receive as incentives or reinforcements when interacting with the app.

##### Barriers to Use of an App

Despite general interest in using an app that could help them to achieve their personal goals, participants reflected on common barriers to app use as a result of their prior use of other apps, and anticipated use of a new app with a new app. Consistent with the facilitators participants described the failure of the app to bring new insights and patronizing comments or features as barriers to use. Participants noted three additional barriers including a carry over-effect, (wherein participants feared a generalization of prior negative experiences with technology), notifications without proper context or personalization, and preferences for in person support.

Many participants described a carry-over effect that involved negative bias toward apps based on past experiences with ineffective digital tools. Further these biases would need to be disconfirmed in early interactions, since participants would have little tolerance for the use of resources that were not helping. On this several participants expressed concerns that an app would provide them with ineffective or generic, recommendations that they had already received elsewhere. For example, one participant described:

“*Well, it's really like the social media stuff that I was talking about earlier … and I have kind of put that, well it's always going to be like that or going onto Google, it's giving only things that don't really help. In my mind, I'm just kind of – I am assuming like every app and everything like that is going to be oh, just write down your stuff or oh, do a little color sheet or something”* (P4).

Similarly, another participant described prior use of an app that supplied stale suggestions, “*I just kept seeing a lot of the same information and suggestions for a long time. And I think, at that point I had known that they didn't work or apply to me very well, and they worked for other people. I think I was kind of like, “Well, I've seen all there is”* (P7). This failure of apps to bring new insight was the most commonly referenced barrier and cause for past disengagement.

In addition to fears of an app bringing more of the same (or content they already have access to online), a common anticipated barrier was that app-based communication (especially in the form of notifications) could be belittling or patronizing. Participants were particularly concerned that an app could make them feel childish if it congratulated them on certain things or was too “cutesy” in its presentation of material. On this, one participant described:

“*I don't use any of the tracking apps or anything like that. I know a lot of people I do. I never got – it felt too self-congratulatory for me to get myself to use one. –It's like, “Good job. You didn't do this, or this, or that for three days. Good job.” It's like I'm not a baby. I don't need to be congratulated for doing something that like nobody else here is doing.”* (P11)

While this participant describes these types of encouragements as a cause for disengagement, others saw them as potentially incentivizing. There were similarly mixed feelings on whether the digital tool should include notifications, or “pull elements” that would initiate interactions in moments when participants were not directly engaged with the app. Though most participants understood the utility of notifications to keep them engaged, motivate them toward their personal goals, and increase self-awareness, they also cautioned that this type of feature could become annoying, burdensome, or exacerbate feelings of guilt for not achieving their goals. As an example of the complicated nature of participant feelings around notifications, one participant described:

“*So, My first Instinct Is to say, “No Notifications,” but Then My Other Feeling Is, if I'm not Regularly Using It or Regularly Thinking About It, I'd say I'd Go six Months Without Self-Harming and Completely Forget About the app on My Phone and Then Experience an Urge, Chances Are I'm not Going to Think to use the app Because I Haven't Used It in so Long or Thought About It and It Hasn't Reminded Me to Check in. So, That's a Really Tough Question. I'm not sure” (P5)*

This participant's comment additionally reflects the perceived benefit that could be derived from the app serving a purpose in periods of more frequent NSSI episodes as well as periods when NSSI was not at the forefront of their concerns.

Notifications related to NSSI behavior could be counterproductive, making some participants feel more shame, guilt, or disappointment in themselves, or activate a competitive drive causing them to injure more frequently. Indeed, some participants felt that check-ins that were specifically tied to their NSSI activity (e.g., have you self-injured today?) or tied to an “app for self-injury” might increase thoughts of NSSI. On this one participant said: “*My first instinct is to say, if I'm getting messages or notifications, I'm more likely to start thinking, and I'm not thinking about self-harm at all, and then I get a notification from an app that has to do with self-harm, I'm going to start thinking about self-harm” (P5)*. Though research has shown that studies utilizing such prompts on suicide and self-harm through ecological momentary assessment do not increase likelihood of harm (39–43), this participant's comment highlights the importance of meeting participants where they are at and respecting their needs.

Finally, in a small number of cases participants expressed a preference for in person support and did not feel that an app would be a good solution for them at this point in time. For example, one participant felt interpersonal relationships were key to her recovery. She described that if something was going to help her with self-management, “*It'd definitely be other people in my life and not a technology thing or anything related to that”* (P2). However, even in these cases participants recognized this as a deeply personal preference and noted the potential usefulness of an app for others who did not have access to such supports or were disinterested in in-person support.

## Discussion

The current study contributes to the literature a more nuanced understanding of the self-management practices of young adults with lived experiences of self-injury, as well as their current and desired use of technology to support these practices. While at a high-level many of these findings are consistent with existing literature on the NSSI recovery, they also extend our understanding of young adults' needs and contribute new insights on how they would like for their needs to be addressed within the design of technologies to support them.

We found that technology use was implicit in many of the activities participants endorsed to reduce NSSI thoughts and urges. Participants commonly accessed NSSI information and support through the internet, even if the usefulness of such online engagement shifted with time. Our findings both highlight young adults' interest in, and willingness to try, a digital intervention (like an app) to support them in reaching their mental health goals and call attention to factors that could enhance or impinge upon the utility of such a tool. In the sections that follow we describe findings with implications to guide the design of digital interventions for this population.

### Person-Centered Design Flexibility for NSSI

Despite interest in trying a digital tool to support NSSI self-management, many participants described that they would approach the app with biases from prior experiences with digital resources. This finding underscores the importance of designing to enhance early engagement and satisfaction, prioritizing user needs and goals early on, and making sure that users receive feedback that is personally relevant.

While personalization, attuning to a clients' needs and motivations, is an inherent part the therapeutic process, it is challenging to accomplish within fully (or partially) automated digital tools. The variability participants' expressed in terms of goals and their experiences of NSSI aligns with a shift toward a person-centered understanding of recovery from NSSI (44–47), and adds complexity to this design challenge. To address this challenge, we advocate for *design flexibility*, which can enable digital interventions to be reconfigured to meet the needs of the user *via* a person-centered approach.

Historically flexibility was discussed in the field of engineering in terms of the ability of a system or product to change and adapt to a range of conditions (48), and accommodate novel future uses (49). In the context of digital intervention design, flexibility includes considerations of the intervention approach, interactive features, and temporal sequencing of content and contact.

Approach flexibility refers to the ability of the tool to frame and present content in a way that is appropriate and acceptable to users. This includes a consideration of user needs, goals, and motivation, and is necessary to mitigate early discouragement and disengagement with the tool. While the psychotherapeutic approach that informs the digital tool (e.g., DBT, CBT) should remain consistent across tool instantiations (so as to not jeopardize the integrity of the intervention model) the way content is delivered should be flexible to account for individual needs. This may include tailored sequencing of materials to prioritize areas for personal growth. For example, during onboarding an app could collect information on participant's NSSI history, and use of strategies to provide a sequence of strategies that build upon their strengths, and fit with their unique profile.

Individual experiences of NSSI recovery are nuanced and often include other facets of well-being such as healthy relationships, daily functioning, and acceptance (44). Consistent with this fact, and with other design research with young people who self-injure (18), our findings revealed a desire for a holistic or transdiagnostic approach to mental health intervention. Though many participants desired features that would specifically target emotional and behavioral patterns connected to NSSI, there was also interest in intervening and working with symptoms that could exacerbate NSSI, such as depression and anxiety, and improving self-care. A flexible delivery format could recommend NSSI-specific resources when NSSI is particularly salient in daily life, while also supporting broader goals around improved well-being when other life challenges are at the forefront. This type of flexibility would need to be guided by user input and may involve passive sensing when this technology is better developed for NSSI.

Interactive flexibility refers to how a user would like to interact with the app, including frequency of contact and language use if there is a dyadic component. Such flexibility can be accomplished by allowing users to adjust the frequency of use by choosing notification timelines that feel appropriate for their unique threshold of contact. Additionally, though all participants reported some desire to shift away from self-injury over time, albeit with expressed ambivalence, the language participants used to talk about their goals reflected different orientations toward NSSI as a target of intervention. As in prior work (45, 47), some participants focused on NSSI cessation while others focused on reducing the frequency and severity of the behavior, or the use of other coping strategies that would make NSSI less needed. Digital tools should have the appropriate lexicon to incorporate person-centered language to best reflect and support young people's needs. Along these lines, a text-messaging intervention developed with input from adolescents with NSSI enabled users to author self-efficacy messages which were stored and then sent to them during pre-determined times throughout the day (50). This design choice gave users autonomy over when and what messages they would receive, and incorporated their language in the design.

In addition to approach and interactive flexibility, apps must be responsive to shifting needs within an individual's own recovery. This refers to temporal flexibility, which may include allowing users to set multiple short- and longer-term goals that can be adjusted as their needs shift. Designing digital tools with logic patterns and inputs that allow individuals to adjust criteria about their goals, their readiness to change their behavior, and their need/desire for contact (e.g., notifications) over time, is one way to protect against an app feeling generic. Another option may be to use automated methods to detect shifts in language (e.g., such as ambivalence) to adapt recommendations over time.

In sum, design flexibility both in terms of individual goals and where individuals are at in their NSSI recovery trajectory were desired.

### Opportunities for Tracking to Increase Self-Knowledge and Improve Recommendations

There was unanimous support for the value of an app connecting users with coping strategies tailored to their unique presentation of NSSI. This is not unsurprising as enhanced self-knowledge and tailored recommendations may increase the likelihood that an individual is able to use a coping strategy that works for them in moments of distress, and subsequently improve self-efficacy. Participants described many functions for their NSSI behavior, as well as different triggers, and activities they tried to reduce or manage these triggers. As such, generic advice was not generally thought to be useful. Many participants were excited about the possibility of an app that could improve upon traditional recommendations found in online forums or from other informational resources (e.g., mindfulness). There was interest in the use of tracking patterns and use of coping skills either through self-report monitoring or passive sensing to detect patterns and provide feedback that could increase self-knowledge and capacity to regulate over time. This desire for tracking is consistent with recommended therapeutic techniques like chain analysis from DBT (51), as well as antecedent-behavior-consequences (ABC) exercises from behavioral therapies (52).

Despite the perceived value of tracking, participants expressed reservations on the tracking of self-injury behaviors, as in other work (29). Participants wanted to become more aware of patterns that might indicate that they were at risk of declining mental health but many did not feel that inputting specifics about self-injury would be useful. Since self-injury recovery can include shifts in NSSI thoughts and urges, in addition to behaviors, as well as other mental health symptoms, future work should identify which aspects of recovery participants feel most comfortable tracking. Passive sensing was suggested as a way to reduce participant burden, while also providing helpful insights on moments of worsening mental health. However, there are currently technical limitations to what can currently be sensed reliably (e.g., mood, urge), and passive metrics relevant for NSSI is an area for future work.

In addition to better understanding their behavior, participants wanted to act on these new insights, especially in moments of acute distress. This finding aligns with past work (32, 33), and emphasizes a need for the technology to be able to engage the individual user in direct actions and to provide scaffolding to help their motivation. For example, rather than providing a space for participants to engage in journaling, prompted journaling where a user is guided through exercises to reflect on patterns or feelings may be more engaging and impactful.

Finally, most participants relied on avoidance-based coping strategies (e.g., distraction), which had some short-term efficacy but did not work reliably and left them feeling unsatisfied. While features that could help users distract were desired, a digital tool may be help users acquire and practice strategies with longer-term efficacy. For example, approach strategies, like problem-solving, have been empirically linked to less NSSI risk (53). Thus, digital tools for this population may provide support to address in-the-moment needs and short-term goals, and also provide exposure to approach-oriented strategies that have been connected to better NSSI outcomes.

### Benefit to Incorporating Peers and Social Supports Mindfully

Our findings also provide new insights on how and why peer support was helpful for participants, and how their desire for peer support shifted over time. For many participants online peer support was especially helpful when they were younger or earlier in their recovery process. However, most participants felt that after a period of using social media or peer support forums the advice they received became stale or they noticed ways the content shared was harmful, or not conducive to their recovery. Despite noting that this format of peer support lost some benefit over time, participants still reflected on the value of knowing that they were not alone, the validation they received, and increased understanding of their own struggles through shared stories. One solution participants described to circumvent potential harms of an interactive interface with other individuals who struggle with NSSI, could be through sharing content and peer stories.

Notably, peer stories were imagined as a means to combat feelings of loneliness and provide participants with validation, but they were also cited as a data source that could be of use to improve that app-based suggestions would resonate with individual users with similar characteristics (e.g., gender, age, LGBTQ^+^) and experiences (e.g., trauma, family conflict). That is, participants felt that peer stories could be used to inform algorithms within the app to tailor recommendations based on the likelihood of a certain strategy or recommendation being relevant to a user. One way to do this may be to crowdsource peer stories and infer distinct user profiles. While there are limits to this approach, future work may wish to consider whether there are distinct profiles of individuals that may benefit from specific skills.

Finally, while the focus of this study was on self-management, opportunities for a self-management technologies to assist individuals connecting with a more robust support system, including informal and formal resources, should not be ignored. The fact that so many participants commented on social and interpersonal supports as relevant to their self-management is consistent with the broader literature on NSSI recovery (54, 55). The goal of developing a digital intervention to support young people in their recovery is not for the tool to be the only solution, or the only support. These interventions can be designed to increase both the users' ability to self-regulate, as well as to improve their understanding of other external supports and their literacy around how and when to reach out to others for support. In line with this, and the idea of design flexibility, app developers may consider ways that a tool can be reconfigured to incorporate feedback and involve interactions with an individuals' broader support system. This may include the ability to generate useful feedback for users to bring to therapy, or share within other contexts where they may seek support around mental health or self-injury.

### Limitations

Despite the strength of this study in engaging with young people with lived experience of self-injury, there are also limitations. The findings from this study do not likely represent the experiences or opinions of all young adults who engage in NSSI. First, participants in our study were recruited from online venues and willingly completed interviews focused on a digital intervention. Thus, they may have been both more inclined to online resources, and more interested in using a digital resource as part of their efforts to manage their self-injury and improve mental health. Secondly, our sample is limited in terms of racial and ethnic diversity. While we had broad representation in terms of gender, 80% of participants were white and no participants were Hispanic. Therefore, our results may not generalize to other more diverse samples and future work should consider ways of engaging young adults from minoritized racial and ethnic groups. Lastly, while our recruitment strategy was focused on individuals who are not currently engaged in mental health treatment, we note that most of our participants were at one time engaged with mental health services. Participants did not specify whether the mental health treatment they received was for NSSI specifically, and all participants were not engaged in mental health treatment at the time of interviews. There are likely differences in the needs of individuals who have experience with therapy, and who have been exposed to coping skills in the context of therapy, and the needs of individuals who have no history of mental health treatment. Given the importance of creating accessible resources for those who have never made contact with treatment services for NSSI, future research should explore how the needs of these individuals differ from those with some treatment history, and implications for engaging with these populations.

## Conclusion

This study advances our understanding of how young adults with NSSI understand their behavior and engage in self-management, the role technology plays in self-management, and their desired use of technology to support them in this process. We found that lived experiences of NSSI are complex, making a one-size-fits all digital tool not just impractical but also likely ineffective. Participants expressed varied experiences with NSSI which impacted their goals, intentions, and needs. However, in sharing their experiences participants offered several possible design solutions that would facilitate the flexibility and autonomy needed to make a resource capable of supporting their shorter- and longer-term goals. These findings have practical implications for the design of digital tools to support this population, and contribute to our understanding of individual experiences of, and with, self-injury recovery.

## Data Availability Statement

The datasets presented in this article are not readily available because this study involves qualitative data from Interviews that is difficult to anonymize. Requests to access the datasets should be directed at: kaylee.kruzan@northwestern.edu.

## Ethics Statement

The studies involving human participants were reviewed and approved by Institutional Review Board at Northwestern University. The patients/participants provided their written informed consent to participate in this study.

## Author Contributions

KK, DM, and MR contributed to conception and design of the study. KK conducted interviews, analyzed data, and wrote the first draft of the manuscript. DM and MR provided feedback throughout study execution. All authors contributed to manuscript revision, read, and approved the submitted version.

## Funding

This work was funded by the National Institute for Mental Health (T32MH115882, R34MH128410, and P50MH119029).

## Conflict of Interest

The authors declare that the research was conducted in the absence of any commercial or financial relationships that could be construed as a potential conflict of interest.

## Publisher's Note

All claims expressed in this article are solely those of the authors and do not necessarily represent those of their affiliated organizations, or those of the publisher, the editors and the reviewers. Any product that may be evaluated in this article, or claim that may be made by its manufacturer, is not guaranteed or endorsed by the publisher.
