# Supplementary material for: How Technologies Can Support Self-Injury Self-Management: Perspectives of Young Adults With Lived Experience of Nonsuicidal Self-Injury
Source: Front Digit Health. 2022 Jun 29;4:913599. doi: 10.3389/fdgth.2022.913599 (PMC9278014; doi:10.3389/fdgth.2022.913599)
Supplement: Supplementary file 1 [file Table_1.DOCX]

**Interview protocol (60 minutes)**

**<introduction / consent >**

**I’d like to start by asking you about how you manage your mental health and well-being in daily life.**

- Could you tell me a little more about what you do when you’re struggling with your mental health (e.g., feeling low, anxious, depressed)?
- Have you tried anything in an effort to manage your mental health?
  - If yes:
    - What helps?
    - What hurts or doesn’t help?
  - If no:
    - Are there specific reasons why you haven’t tried anything yet?
    - Do you wish to in the future?
- Do you have any current goals related to your mental health?
  - If so, what are those goals?

**Now I’m going to ask you more specifically about what you’ve tried to manage self-injury urges.**

- Could you tell me a little more about what you do when you have self-injury thoughts or urges?
- Have you tried anything to manage your self-injury thoughts or urges?
  - If yes:
    - What helps?
    - What hurts or doesn’t help?
  - If no:
    - Are there specific reasons why you haven’t tried anything yet?
    - Do you wish to in the future?
- Do you have any current goals related to your self-injury thoughts and behaviors?
  - If so, what are those goals?

**Now, I’m curious to learn more about how technologies fit into your overall self-management routine…**

- Do you use any technologies to support your mental health and well-being?
  - If yes:
    - Could you tell me a bit about what you use?
    - When do you use these technologies?
    - Does it help? Why or why not?
  - If no:
    - Have you ever used any technologies to support your mental health or well-being?
      - If yes:
        - Could you tell me a bit about what you’ve used?
        - When did you use these technologies?
        - Did it help? Why or why not?
        - What made you stop using [technology]?
      - If no:
        - Why not?
- Do you use any technologies to regulate or help you manage self-injury urges or thoughts?
  - If yes:
    - Could you tell me a bit about what you use?
    - When do you use these technologies?
    - Does it help? Why or why not?
  - If no:
    - Have you ever used any technologies to regulate or help you manage your self-injury?
      - If yes:
        - Could you tell me a bit about what you’ve used?
        - When did you use these technologies?
        - Did it help? Why or why not?
        - What made you stop using [technology]?
      - If no:
        - Why not?

Next, I’m going to ask you about some specific uses of technology. Some people use technologies like apps, social media or online communities or forums to **talk about things they are going through** or to **seek support**.

- Have you ever used technologies to talk about, or seek support, for **mental health or self-injury**?
  - If yes:
    - What technologies have you used to talk about, or seek support for, your mental health or self-injury?
    - Was this for self-injury specifically?
    - Do you currently use these technologies?
    - What do [did] you do? When do [did] you use these?
    - Do [did] you find this helpful? Why or why not?
  - If no:
    - Why not?

Some people use technologies to **access information about mental health or self-injury, to learn more, and to seek out alternative coping strategies.**

- Have you ever used technology to get information about how to manage your mental health?
  - If yes:
    - What technologies have you use to learn more?
    - What types of information did you seek?
    - Was this for self-injury specifically?
    - Do you currently use these technologies?
    - Did you ever used that information to manage your mental health / self-injury?
  - If no:
    - Why not?

**Are there any ways you’d imagine wanting to use technologies, such as an app, to support you in your self-injury self-management?**

- What types of technologies would you want to use?
- *What would you want it to do?*
- *Would you want to use it alone or with a coach or to interact with others?*
- What types of content, or information, would you want this technology to have?
- How would you like to interact with this technology (e.g., daily, weekly, through messaging, etc.)?

End of interview (thank you, ask for full name/email/physical address/phone# for $25 compensation)
